# Healthcare Cost and Utilization before and after Diagnosis of *Pseudomonas aeruginosa* among Patients with Non-Cystic Fibrosis Bronchiectasis in the U.S.

**DOI:** 10.3390/medsci5040020

**Published:** 2017-09-23

**Authors:** Christopher M. Blanchette, Joshua M. Noone, Glenda Stone, Emily Zacherle, Ripsi P. Patel, Reuben Howden, Douglas Mapel

**Affiliations:** 1 University of North Carolina at Charlotte, Department of Public Health Sciences, Charlotte, NC 28223, USA; cblanche@uncc.edu (C.M.B.); jnoone1@uncc.edu (J.M.N.); ezacherl@uncc.edu (E.Z.); ripsi.patel@uncc.edu (R.P.P.); rhowden@uncc.edu (R.H.); 2 Precision Health Economics, Huntersville, NC 28078, USA; 3 Grifols, Durham, NC 27709, USA; 4 University of New Mexico College of Pharmacy, Albuquerque, NM 87131, USA; dmapel@comcast.net

**Keywords:** bronchiectasis, *Pseudomonas aeruginosa*, healthcare costs, healthcare utilization, exacerbations, chronic lung disease, bronchial inflammation

## Abstract

Non-cystic fibrosis bronchiectasis (NCFBE) is a rare, chronic lung disease characterized by bronchial inflammation and permanent airway dilation. Chronic infections with *P. aeruginosa* have been linked to higher morbidity and mortality. To understand the impact of *P. aeruginosa* in NCFBE on health care costs and burden, we assessed healthcare costs and utilization before and after *P. aeruginosa* diagnosis. Using data from 2007 to 2013 PharMetrics Plus administrative claims, we included patients with ≥2 claims for bronchiectasis and >1 claim for *P. aeruginosa*; then excluded those with a claim for cystic fibrosis. Patients were indexed at first claim for *P. aeruginosa* and were required to have >12 months before and after the index *P. aeruginosa*. The mean differences in utilization and costs were assessed using paired Student’s *t*-tests for statistical significance. Mean total healthcare costs per patient were $36,213 pre-*P. aeruginosa* diagnosis versus $67,764 post-*P. aeruginosa*, an increase of 87% (*p* < 0.0001). Inpatient costs represented the largest proportion of total healthcare costs post-*P. aeruginosa* (54%) with an increase of four hospitalizations per patient (*p* < 0.0001). NCFBE patients with evidence of *P. aeruginosa* incur substantially greater healthcare costs and utilization after *P. aeruginosa* diagnosis. Future research should explore methods of earlier identification of NCFBE patients with *P. aeruginosa*, as this may lead to fewer severe exacerbations, thereby resulting in a reduction in hospitalizations and healthcare costs.

## 1. Introduction

Non-cystic fibrosis bronchiectasis (NCFBE) is a chronic disease characterized by a cyclical process of recurrent infections, pulmonary inflammation and structural damage to the airways. Prevalence of NCFBE in the U.S. has been estimated to be 139 cases per 100,000 [1]. Frequent exacerbations significantly contribute to higher morbidity and mortality among NCFBE patients, as well as increased health care utilization and costs [2]. NCFBE places a significant burden on the healthcare system with an estimated average incremental cost to a U.S. health plan of $4862–$6593 per patient per year (2005 USD) [3]. In the first year after diagnosis, patients cost an incremental average of $2300 per patient per year (2009 USD) [4]. NCFBE patients with an additional diagnosis of *P. aeruginosa* infection may experience even greater burden.

Between 50 and 80% of NCFBE cases are idiopathic and the remainder secondary to post-infection resulting from underlying disease, obstruction or other airway related injury [4,5,6]. The pathophysiology of NCFBE leads to disruption and dysfunction of the normal epithelial barrier, consequentially allowing pathogens to colonize airways and cause frequent infectious episodes [7,8]. In NCFBE patients, *P. aeruginosa* is the most prevalent cause of infection, which leads to more hospital admissions than other NCFBE-related infections [2,9,10,11]. Prevalence of *P. aeruginosa* among NCFBE patients has been reported to be as high as 15–58%, however many of these estimates are based on single centre investigations and some studies include cystic fibrosis patients in their prevalence calculations [10,12,13,14,15]. There has not been a large nationwide assessment of diagnostic patterns in the U.S. making it difficult to generalize prevalence rates. Given these circumstances, actual estimates of *P. aeruginosa* prevalence in the U.S. are not well understood. 

NCFBE patients with *P. aeruginosa* infection and frequent exacerbations experience accelerated decreases in lung function, reduced quality of life, increased length of stay and risk of hospitalization, and both higher morbidity and mortality [2,7,9,10,11,12,15,16,17,18,19,20,21,22]. It is well recognized that NCFBE patients with *P. aeruginosa* infections have more frequent exacerbations and accelerated rapid disease progression than NCFBE patients without *P. aeruginosa* [2,9,11,21,23]. Other common infections in NCFBE patients include *Haemophilus influenzae*, nontuberculous mycobacteria, and *Moraxella catarrhalis*, but these infections are not reported to be as prevalent as *P. aeruginosa* [24,25]. 

Management is complicated by the fact that no gold standard exists for the diagnosis, management, and treatment of *P. aeruginosa*. Disease management requires obtaining sputum cultures and treatment options vary from inhaled antibiotics to dual anti-pseudomonal therapy [24,26]. While it is recognized that *P. aeruginosa* is among the most common pathogens responsible for nosocomial infections, a plethora of questions remain unanswered in determining the optimal therapeutic treatment in patients with NCFBE [9]. A patient’s management of *P. aeruginosa* may include the use of several antibiotics due to antibiotic resistance and treatment ineffectiveness, perhaps contributing to the higher costs seen following *P. aeruginosa* diagnosis in the present study [9,27,28].

Currently, no studies have effectively evaluated the impact of *P. aeruginosa* on healthcare costs and utilization related to NCFBE in the U.S. Given the significant impact of *P. aeruginosa* on clinical outcomes in NCFBE, it is anticipated that *P. aeruginosa* diagnosis increases economic burden on the healthcare system versus NCFBE without *P. aeruginosa*. Therefore, the purpose of this study is to evaluate the healthcare costs and utilization pre- and post-*P. aeruginosa* diagnosis among commercially insured NCFBE patients in the U.S.

## 2. Materials and Methods

To complete this analysis, we used 2007–2013 claims data from the PharMetrics Plus database. This database provided pooling of adjudicated medical and pharmacy claims for over 100 million patient lives from more than 250 health plans across the U.S. As an administrative claims dataset, PharMetrics includes inpatient and outpatient diagnoses in International Classification of Diseases-Ninth Revision-Clinical Modification (ICD-9-CM and ICD-10) format, procedures in Current Procedural Terminology-Fourth Edition and the Healthcare Common Procedure Coding System and prescription records. Also included in the data are demographic variables, product and insurance type, provider specialty, and dates inclusive of plan enrollment [29].

NCFBE patients were originally identified by the presence of at least two claims with the ICD-9 code for bronchiectasis (494.XX) and the absence of any claims for cystic fibrosis (277.XX). To further ensure proper patient selection, the claims for bronchiectasis were required to be at least 90 days apart. Patients were required to have 12 months of claims experience before and after the index *P. aeruginosa* claim for the assessment of healthcare cost and resource utilization. Patients were stratified by *P. aeruginosa* diagnosis at any point during the study period using ICD-9-DM codes for 482.1 (pneumonia due to *Pseudomonas)* and 041.7 (pseudomonas). Patients were indexed at their first *P. aeruginosa* claim. 

Baseline characteristics of all patients were collected from the one-year pre-period and included Elixhauser comorbidity conditions, age, and gender. Mean and median total and disease-related 12-month post-index disease related healthcare utilization and costs were evaluated to measure the burden of *P. aeruginosa* diagnosis on NCFBE patients [30]. Post diagnosis all-cause healthcare cost measures included: mean total annual healthcare, hospitalization, emergency room, pharmacy, and physician office costs for patients with a primary or secondary claim for *P. aeruginosa*. Post diagnosis *P. aeruginosa*-related costs were those with a primary claim for *P. aeruginosa*. Costs outside these categories were classified as ‘other costs’, and included, but were not limited to: nursing home, home health, ambulance services, rehabilitation, durable medical equipment, and laboratory costs. Post diagnosis all-cause healthcare utilization measures included mean annual hospital, emergency room, physician office and pharmacy visits. All statistical analysis was conducted using SAS version 9.4 (SAS Institute, Cary, NC, USA). All tests were conducted assuming a two-tailed test of significance and alpha level set a priori at 0.05. Demographic and medical characteristics for the cohort were measured using counts and percentages for categorical variables and measures of central tendency for continuous variables. Characteristics were presented by total sample. Negative binomial models were used to assess factors associated with counts of events to include inpatient hospitalizations, outpatient events, physician office visits, emergency room visits, and total pharmacy utilization while controlling for covariates. A paired Student’s *t*-test for statistical significance was conducted to determine the mean difference in utilization and costs. Model fit characteristics were assessed and assumptions of the model were upheld.

## 3. Results

Initial cohort selection identified 23,740 patients with NCFBE with 716 having been diagnosed with *P. aeruginosa*. Females accounted for over half the NCFBE with *P. aeruginosa* population (60.47%). The majority of patients were 50+ years (88.83%), with 7.54% in the 18–49 and 3.07% in the 0–17 year age ranges. Chronic obstructive pulmonary disease (COPD), uncontrolled hypertension, and cardiac arrhythmias were the most prevalent comorbidities among NCFBE patients with *P. aeruginosa* (86.45, 49.58, and 28.63% respectively) (See Table 1). 

**Figure 1:**
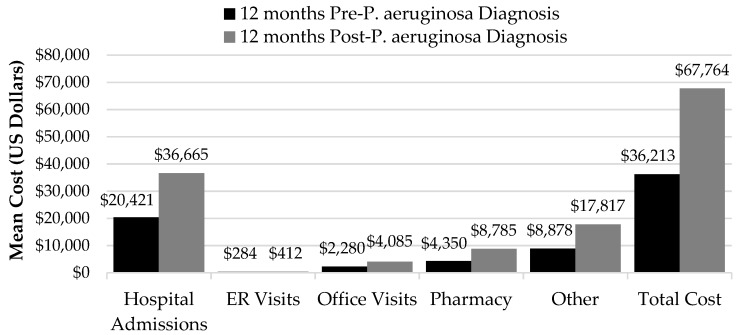
All-cause healthcare costs (pre- vs. post-*P. aeruginosa*).

When comparing all-cause healthcare costs pre- versus post-*P. aeruginosa*, mean total per patient costs pre- *P. aeruginosa* were $36,213 ± 132,270 on average compared to $67,764 ± 154,750 post-*P. aeruginosa*, equating to an 87% or $31,551 increase post-diagnosis (*p* < 0.0001). Mean hospital costs accounted for the largest proportion of healthcare costs both pre- and post-*P. aeruginosa* diagnosis ($20,421 ± 125,986 pre- and $36,665 ± 135,836 post-*P. aeruginosa*; *p* = 0.0004), followed by other costs ($8878 ± 24,276 pre- and $17,817 ± 42,036 post-*P. aeruginosa*; *p* = 0.0163), pharmacy costs ($4350 ± 8084 pre- and $8785 ± 12,162 post-*P. aeruginosa*; *p* < 0.0001), office visit costs ($2280 ± 4471 pre- and $4085 ± 7748 post-*P. aeruginosa*; *p* < 0.0001), and emergency room (ER) visit costs ($284 ± 1305 pre- and $412 ± 1382 post-*P. aeruginosa*; *p* < 0.0001). Post-*P. aeruginosa* diagnosis pharmacy and other costs were over two times higher than pre-*P. aeruginosa* costs (202 and 201% higher, respectively) (See Figure 1).

Total mean *P. aeruginosa*-related healthcare costs were $18,407 (±58,450), of which hospitalizations again accounted for the majority of costs ($16,935 ± 58,269). Mean other *P. aeruginosa*-related costs were $1206 (±3665), mean office visit costs were $256 (±1560), and mean ER visit costs amounted to $9 (±166). 

**Figure 2:**
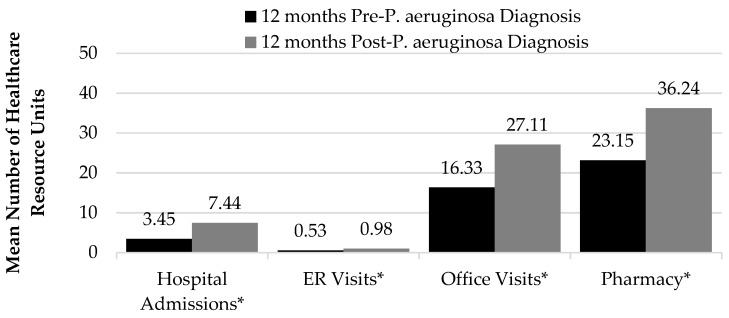
Healthcare utilization (pre- vs. post-P. Aeruginosa; * p < 0.0001).

Mean hospital admissions increased by 115.63% from the pre- to post-*P. aeruginosa* period (3 ± 13 pre- vs. 7 ± 15 post-*P. aeruginosa*; *p* < 0.0001). Mean ER visits increased by 82.51%, office visits by 66.04% and pharmacy visits by 56.50% (*p* < 0.0001 for all) (See Figure 2).

## 4. Discussion

Healthcare cost and resource utilization increased substantially for NCFBE patients after *P. aeruginosa* diagnosis, which is not surprising from a clinical perspective. However, this study is the first to describe an incremental cost post-*P. aeruginosa* diagnosis. Even though the cost differences found were significantly higher post-*P. aeruginosa*, it is possible that the true incremental cost may be substantially higher if *P. aeruginosa* was present but undetected in the pre-*P. aeruginosa* diagnosis period. Previous reports evaluating the economic burden of NCFBE have suggested that management of this disease is associated with high costs to the U.S. healthcare system, but have not evaluated the impact of *P. aeruginosa* diagnosis on these costs. One study estimated that in 2013 between 340,000 and 522,000 persons in the U.S. were receiving treatment for NCFBE during that year [1]. Clinically speaking, *P. aeruginosa* contributes to the chronic cycle of infection and inflammation in NCFBE which is associated with exacerbations, increased symptoms, decline in lung function, reduced quality of life and ultimately, increased healthcare resource use [2,7,9,10,11,12,15,16,17,18,19,20,21,22]. Previous studies have identified three factors associated with disease progression which results in functional limitations in NCFBE patients: (1) *P. aeruginosa* in the sputum, (2) frequency of severe exacerbations, and (3) systemic inflammation. All three of these factors are a major component of the increased economic burden of *P. aeruginosa* on NCFBE patients. However, whether the presence of *P. aeruginosa* among NCFBE patients resulted in increased costs was not addressed in these previous analyses [3,4]. 

Costs beyond NCFBE were mostly attributed to acute healthcare services such as hospitalizations and emergency room visits. We found that resource utilization, including hospital, ER, office, and pharmacy visits, among *P. aeruginosa* patients increased by 115.63, 82.51, 66.04, and 56.60%, respectively, from the pre- to post-*P. aeruginosa* period. To put these findings in context, in a longitudinal retrospective observational cohort study by McDonnell et al. [2], NCFBE patients with *P. aeruginosa* experienced higher hospitalization rates than their non-*P. aeruginosa* infected counterparts. However, McDonnell et al. did not include the economic burden of PA in their analysis [2,3,4]. In our economic analysis, we found an 87% increase in in-patient costs post-*P. aeruginosa*. 

PA infection has been identified as a marker of more severe disease, which leads to higher costs. Furthermore, *P. aeruginosa* is a component of two bronchiectasis severity scoring systems, the Bronchiectasis Severity Index (BSI) and the FACED (forced expiratory volume in one second (FEV1), age, chronic colonization, extension, and dyspnea) score. In a study by Goeminne et al., *P. aeruginosa* in addition to a variety of demographic and clinical factors, was associated with higher mortality in NCFBE [15]. This is consistent with other studies, which found *P. aeruginosa* to be associated with higher mortality, as well as greater airflow obstruction, increased severity of disease and poorer quality of life among NCFBE patients [13,22]. Given that *P. aeruginosa* is accepted as a marker of disease severity, and assuming patients with more severe disease require more medical attention, it is not surprising that the current analysis found increased healthcare resource use and costs in the year following diagnosis. 

The delayed time to *P. aeruginosa* diagnosis and treatment process of NCFBE patients with *P. aeruginosa* may also be responsible for increased healthcare utilization and costs found in the current analysis. The management of *P. aeruginosa* in NCFBE patients varies per patient and is largely dependent on patient history and clinician preference. Comprehensive outpatient follow-up care is required as treatment failures due to antibiotic resistance and exacerbations may occur. Patients with more severe exacerbations, such as hypoxemia or respiratory distress, are often hospitalized. Severe exacerbations that lead to hospitalizations are most troublesome and have the greatest impact on disease progression, healthcare resource utilization, and costs. Further, patients with severe exacerbations may require long-term antibiotic therapy. Clinicians suggest the optimal duration of long-term antibiotic therapy to range from three months to a year, leading to a further increase in healthcare utilization and cost [13,31,32,33]. In combination, all of these factors may be responsible for the higher costs and utilization seen 12 months post-diagnosis in the present study.

With the results of the current study linking *P. aeruginosa* to healthcare use and costs, there needs to be an emphasis on increased rates of testing for NCFBE related infections. Establishing current rates of testing is difficult due to a lack of definitive guidelines. The potential cost of diagnostic testing is significant as there are a number of laboratory and imaging studies, tests, and procedures that may be helpful in confirming a *P. aeruginosa* infection. Testing methods may include: pulmonary function tests, sputum cultures, molecular tests, and antibiotic susceptibility tests [9,34]. Previous reports argue the need for more sensitive testing such as molecular tests in hopes of facilitating early detection of *P. aeruginosa* and thus, increasing the prospect of successful eradication [9]. Further, by identifying more sensitive testing for identifying bacteria responsible for infection, costs associated with multiple testing strategies can be avoided, thereby lowering costs incurred along a patient’s path to a diagnosis.

The current study found the majority of patients to be over the age of 50. This finding is consistent with a previous study that reported the majority of patients to be between the age of 45 and 64 years [4]. It is well understood that increased age is correlated with higher rates of comorbidities versus those who are of younger age. Additionally, comorbidities associated with NCFBE—such as diabetes, chronic lung disease, cardiac arrhythmias, COPD and uncontrolled hypertension—typically lead to more frequent exacerbations as well [26]. The older age of the population combined with the finding that NCFBE patients with *P. aeruginosa* are burdened with many other comorbidities may also account for higher resource utilization and cost found in this study [1,2,3,4].

We acknowledge that the time at which the NCFBE patient acquired the *P. aeruginosa* infection may not have necessarily coincided with the timing of testing and diagnosis of the infection. However, these patients appeared to consume greater healthcare services and incur increased costs following their *P. aeruginosa* diagnosis. Additionally, while claims data is useful in providing a large study population, the potential for misclassification exists. Claims data have the potential for errors and miscoding, which may potentially introduce misclassification bias. Patients may have been incorrectly identified due to this potential error. Another limitation of claims data is that data identifying specific treatments used or patient severity were not available. Therefore, it is not known if patients truly had a chronic infection of *P. aeruginosa* or the claim was for a single isolated incident.

The findings from this study provided insight on the impact of *P. aeruginosa* on healthcare costs and resource utilization related to NCFBE, an area that had been previously unexplored. Management of *P. aeruginosa* may be complicated by multiple obstacles such as lengthy patient diagnostic experience, inconsistent or inappropriate treatment strategies, and more severely progressed disease, which lead to further increases in healthcare resource utilization and economic burden. Future economic analyses should investigate the prevalence, diagnosis, and treatment of *P. aeruginosa* and the implications for patients, payers, and other stakeholders.

## Figures and Tables

**Table 1:** Demographics and clinical characteristics of Non-Cystic Fibrosis Bronchiectasis (NCFBE) patients with *P. aeruginosa*.

	*N*	%
**Total**	716	100
**Mean**		
0–17 years	22	3.07
18-49 years	54	7.54
50+ years	636	88.83
Unknown	4	0.56
**Sex**		
Female	433	60.47
Male	282	39.39
Unknown	1	0.14
**Comorbidities**		
Chronic obstructive pulmonary disease	619	86.45
Arterial hypertension, uncontrolled	355	49.58
Cardiac arrhythmias	205	28.63
Fluid and electrolyte disorders	149	20.81
Diabetes, uncontrolled	148	20.67
Congestive heart failure	118	16.48
Valvular disease	108	15.08
Depression	100	13.97
Hypothyroidism	99	13.83
Weight loss	90	12.57
Rheumatoid arthritis	86	12.01
Peripheral ulcer disease	77	10.75
Tumor	76	10.61
Other neurological disease	73	10.2
Pulmonary circulation disease	69	9.64
Deficiency anemia	64	8.94
Arterial hypertension, controlled	62	8.66
Renal failure	57	7.96
Coagulopathies	42	5.87
Liver disease	41	5.73
Diabetes, controlled	38	5.31
Obesity	35	4.89
Paralysis	29	4.05
Lymphoma	26	3.63
Blood loss anemia	14	1.96
Psychosis	12	1.68
Alcohol abuse	11	1.54
Drug abuse	10	1.4
Peptic ulcer disease	10	1.4
Human immunodeficiency syndrome/acquired immunodeficiency syndrome	1	0.14
